# Practical Departments

**Published:** 1902-08-23

**Authors:** 


### PRACTICAL DEPARTMENTS.

### A NEW MECHANICAL BEDSTEAD.

Many attempts have been made to construct an adjustable mechanical bedstead, which, while allowing for a variety of changes of position for its more or less helpless occupant, shall yet be capable of easy manipulation by the nurse, and at the same time adaptable to modern hygienic requirements in the matter of cleanliness. Too often a complication of mechanism defeats the objects aimed at, and so far bedsteads which require much adjustment have not become popular in hospital wards or private houses. Messrs. J. and A. Carter, who are invariably practical in their methods, have lately designed a new bedstead, the "Surcar," which appears to possess most of the advantages and few of the faults of such appliances. In appearance it is simply a particularly solid bedstead of ordinary make and shape, except that the upright corner pillars are thicker and heavier, being in reality tubular iron frames to allow of the mechanical adjustment. The spring mattress, which forms the foundation, so to speak, is specially made to afford complete and even support to the body; above this comes a stretcher, or "elevating frame," which can either rest upon the mattress or be raised to any required height by means of a handle at the bed foot. The stretcher is composed of an iron frame, across which a strong webbing foundation is arranged in bands, very cleverly fastened by straps, in such a manner that they may be removed, either altogether or in sections, to facilitate the dressing of wounds, or for washing the patient, or for the use of the bedpan. The stretcher works most easily, without vibration, noise, or exertion, so that it can be raised or lowered without any trouble to nurse or invalid.

The frame is further supplied with a back-rest and a leg-rest, the latter allowing for one or both legs to be lowered, as desired. A sitting position is thus attainable, and many changes can be effected with a minimum of inconvenience. Being perfectly plain in design, and every portion of the bed removable, it can be kept quite free from dust, and washed when necessary. The arrangement of the webbing bands on the stretcher appears to be particularly good. Castors might, we think, be added with advantage, at least on two of the feet; this enables the bedstead to be moved if necessary, but prevents it from slipping too easily on a polished floor.

Bedsteads of this description must always be too heavy in make, and too expensive to become general in use, but there are many hospital wards where one such bed would prove of the greatest comfort in special cases; and where money does not matter, and in cases of long and helpless illness, the benefit of a practically designed adjustable frame is incalculable. The "Surcar" bedstead, upon which the makers are to be congratulated, is to be seen at Messrs. J. and A. Carter's showrooms, 6A New Cavendish Street, Portland Place, London, W.

